# A Review of Doses for Dental Imaging in 2010–2020 and Development of a Web Dose Calculator

**DOI:** 10.1155/2021/6924314

**Published:** 2021-12-10

**Authors:** Hawon Lee, Andreu Badal

**Affiliations:** ^1^Montgomery Blair High School, Silver Spring, MD, USA; ^2^Division of Imaging, Diagnostics and Software Reliability, OSEL, CDRH, Food and Drug Administration, Silver Spring, MD, USA

## Abstract

Dental imaging is one of the most common types of diagnostic radiological procedures in modern medicine. We introduce a comprehensive table of organ doses received by patients in dental imaging procedures extracted from literature and a new web application to visualize the summarized dose information. We analyzed articles, published after 2010, from PubMed on organ and effective doses delivered by dental imaging procedures, including intraoral radiography, panoramic radiography, and cone-beam computed tomography (CBCT), and summarized doses by dosimetry method, machine model, patient age, and technical parameters. Mean effective doses delivered by intraoral, 1.32 (0.60–2.56) *μ*Sv, and panoramic, 17.93 (3.47–75.00) *μ*Sv, procedures were found to be about1% and 15% of that delivered by CBCT, 121.09 (17.10–392.20) *μ*Sv, respectively. In CBCT imaging, child phantoms received about 29% more effective dose than the adult phantoms received. The effective dose of a large field of view (FOV) (>150 cm^2^) was about 1.6 times greater than that of a small FOV (<50 cm^2^). The maximum CBCT effective dose with a large FOV for children, 392.2 *μ*Sv, was about 13% of theeffective dose that a person receives on average every year from natural radiation, 3110 *μ*Sv. Monte Carlo simulations of representative cases of the three dental imaging procedures were then conducted to estimate and visualize the dose distribution within the head. The user-friendly interactive web application (available at http://dentaldose.org) receives user input, such as the number of intraoral radiographs taken, and displays total organ and effective doses, dose distribution maps, and a comparison with other medical and natural sources of radiation. The web dose calculator provides a practical resource for patients interested in understanding the radiation doses delivered by dental imaging procedures.

## 1. Introduction

Dental imaging is one of the most common types of diagnostic radiological procedures taken by the average person. Popular dental imaging procedures include intraoral radiography, which has the longest history of use, followed by panoramic radiography, and more recently, cone-beam computed tomography (CBCT) [1]. Intraoral radiography, a simple two-dimensional (2D) projection imaging, is often used to detect periodontal disease and cavities at regular dental check-ups. Panoramic radiography, a more comprehensive 2D image that combines a series of narrow 2D images, has been widely used to provide a wide range of information about the dentition and jaws. Introduced in the late 1990s, three-dimensional (3D) imaging technology, CBCT, offers a comprehensive set of cross-sectional images, the ability of vertical scanning, and real-time intraoperative assessment. All three procedures expose different portions of the head, from small parts of the teeth to the whole lower head, to ionizing radiation. There are concerns about the increasing use of imaging procedures as well as the resulting radiation dose, especially for pediatric patients [2, 3].

Absorbed dose is defined as the energy deposited to a given volume divided by the mass (measured in gray, Gy, in the International System of Units) [4]. Equivalent dose (measured in sieverts, Sv) is derived from the absorbed dose multiplied by the radiation weighting factor, which represents the effectiveness of the biological damage to the exposed tissue. Effective dose (measured in sieverts, Sv) is then derived by adding all equivalent doses multiplied by tissue weighting factors and provides a relative measure of the risk of stochastic effects that might result from irradiation. The most fundamental dose quantity, organ dose, of dental imaging can be obtained through two methods: measurement and computer simulation. First, organ doses can be physically measured with dosimeters placed within anatomy models, called physical human phantoms, that are exposed to dental radiation. Second, organ doses can be calculated through computer simulations where the simulation model of an imaging device is combined with digital anatomy models, called computational human phantoms [5]. Different types of pediatric and adult computational human phantoms are available for dose calculations. Many studies report organ doses from dental imaging procedures estimated by measurement or simulation. However, there are few resources that summarize a variety of data and present the radiation dose with a user-friendly interface.

The current study was intended to provide a practical resource for patients interested in understanding the radiation doses delivered by dental imaging procedures for the period of 2010–2020 and comparison with other radiation sources that are commonly faced in daily life. We established a comprehensive table of organ doses for dental imaging procedures by extracting data from literature and developed a user-friendly web application to present the summarized information.

## 2. Materials and Methods

We obtained articles from PubMed, published after 2010, on organ and effective doses delivered by dental imaging procedures including intraoral radiography, panoramic radiography, and CBCT, and summarized doses by dosimetry method, machine model, patient age, and technical parameters. Monte Carlo simulations of representative cases of the three dental imaging procedures were conducted to estimate and visualize the dose distribution within the head. Finally, we developed an interactive web-based dose calculator to provide easy access to the dental doses and to compare them with other radiation sources commonly faced in daily life.

### 2.1. Literature Search

We searched for articles on organ and effective doses delivered by dental imaging in PubMed (https://pubmed.ncbi.nlm.nih.gov, National Library of Medicine, National Center for Biotechnology Information) available on October 1, 2020, using the following keywords:

- “dental intraoral organ dose” (for intraoral)
- “dental panoramic organ dose” (for panoramic)
- “dental cone beam CT organ dose” (for CBCT)

These keywords brought up 41, 49, and 54 papers (144 in total) for intraoral, panoramic, and CBCT procedures, respectively. We selected papers published after 2010:a total of 81 papers (14, 20, and 47 papers for intraoral, panoramic, and CBCT, respectively) out of 144 papers. The papers that were not written in English (except for non-English articles with dose tables in English) or did not include the dose to bone marrow, brain, salivary glands, and thyroid and effective doses were excluded from the review process. After the exclusion, we finally used 3, 9, and 11 papers providing organ and effective doses for intraoral, panoramic, and CBCT procedures, respectively.

### 2.2. Data Collection

The following data were extracted from the papers:

- Dosimetry methods: simulation or measurement
- Imaging machine model
- Age represented by physical (measurement) or computational (simulation) phantoms: we denoted the age of 35 as the minimum age for all adult phantoms, which is the International Commission on Radiological Protection (ICRP) reference age of adults [6]
- Dose calculation program for simulation studies or dosimeter type for measurement studies
- Beam rotation angle (only for CBCT)
- Imaging protocol
- Dose area product (DAP) (mGy-cm^2^) (for intraoral and CBCT)
- Tube potential (kVp)
- The width and height of field of view (FOV) (cm) (for intraoral and CBCT)
- Effective dose (E) (*μ*Sv)
- Doses to the bone marrow, brain, salivary glands, and thyroid (*μ*Gy)

When a single paper provided multiple dose data in multiple categories, the dose in each category was considered a separate dose set. Simulation and measurement data were analyzed separately when both were reported in a single paper. When an effective dose was missing but organ doses were reported, an effective dose was derived from the organ doses using tissue weighting factors from ICRP Publication 103 [4]: 0.12 (bone marrow), 0.01 (brain), 0.01 (salivary glands), and 0.04 (thyroid). We assumed zero doses for other organs outside the head region in the calculation of the effective dose. The extracted data were tabulated in three detailed tables for intraoral, panoramic, and CBCT, respectively.

To efficiently analyze the doses, we averaged the organ and effective doses over different data sources. As for CBCT, which had more available data for different phantom ages and FOVthan intraoral and panoramic, we further arranged organ and effective doses by phantom age group (children and adults) and/or the area (height *x* width) of FOV (small <50 cm^2^, medium 50–150 cm^2^, and large >150 cm^2^).

### 2.3. Monte Carlo Simulation of Dental Imaging Procedures

We conducted Monte Carlo simulations of intraoral, panoramic, and CBCT imaging procedures by using a computational human head phantom and multimodal imaging-based detailed anatomical (MIDA) [7]combined with a Monte Carlo radiation transport code, MC-GPU [8]. The voxel resolution of the head phantom was 0.5 × 0.5 × 0.5 mm^3^. Key technical parameters for Monte Carlo simulations that were collected from literature are summarized in Table 1. In the case of panoramic imaging, a simplified image acquisition was modeled by concatenating 9,153 simulations with a 1-pixel-wide field of view of 10 × 0.05 cm^2^ instead of the realistic field of view of 10 × 0.2 cm^2^ since the image overlap in real machines could not be reliably simulated. Our simulations had two purposes: to evaluate the proportion of the dose distribution among different tissues during the three imaging modalities and to visualize dose distribution across the head anatomy in the web program.

### 2.4. Development of a Web-Based Dose Calculator

After the summary dose tables were established, we developed a web-based dose calculation program to allow for convenient access to the organ and effective doses and comparison of the dental doses with doses from other radiation sources.

The web program was designed to allow an input from the user for the following parameters: type of imaging modalities, number of image sets, patient age group (child or adult), and size of imaging region, which is the area of the FOV (small <50 cm^2^, medium 50–150 cm^2^, and large >150 cm^2^). The last two parameters were only used for CBCT dose as the dose data for intraoral and panoramic imaging were not enough to be stratified by age and FOV. The user has the option of “I do not know” for the patient age group and FOV, in which case the average dose of the age groups and/or the size of FOV were presented.

Based on the input data from a user, the web application presents the following information:

- Dose delivered to the bone marrow, brain, salivary glands, and thyroid and effective dose; the doses are calculated by multiplying the dose per imaging by the number of image sets inputted by a user. Limited pediatric data points were available for intraoral and panoramic, andonly a total of 22 pediatric data points were extracted for CBCT. Since the data points were not enough to derive age dependency of dose for finer age resolution, we combined the 22 data points for CBCT into the pediatric age group. In the case of CBCT, when the user selects both age group and FOV, age and FOV dependent doses are displayed. FOV-averaged doses are displayed when a user selects “I do not know” for FOV. Age-averaged dose is displayed when a user selects “I do not know” for age.
- 2D and 3D dose distribution for the selected imaging procedures and the fraction of dose delivered to different tissues
- Comparison of the total effective dose (effective dose multiplied by the number of image sets) with that from other radiation sources: 37 *μ*Sv (London-to-New York flight), 100 *μ*Sv (chest X-ray) and 3110 *μ*Sv (annual natural background) [9]

**Figure 1:**
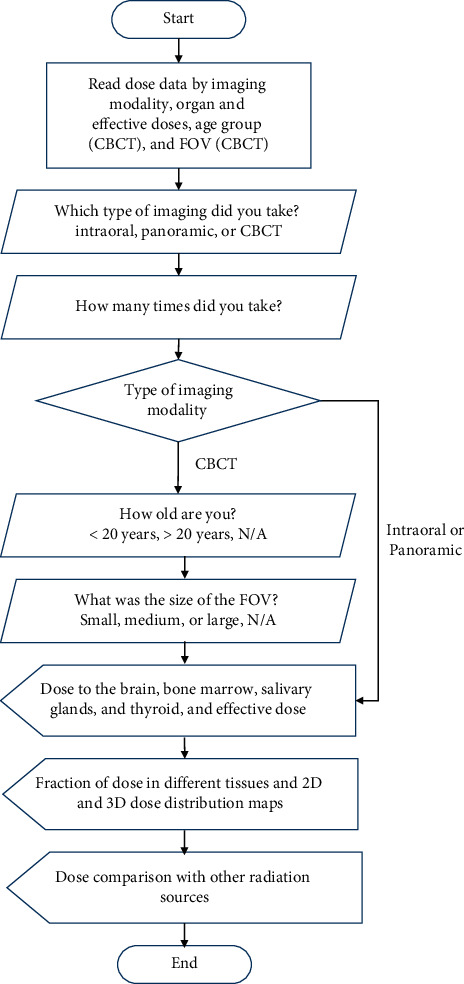
Flowchart of the web application for dental radiation dose calculations and dose display.

The web dose calculator was developed using the commercial cross-platform language, Xojo (Xojo, Inc., Austin, TX). The Xojo development tool provides a graphical user interface-based programming environment to develop multiplatform apps for macOS, Windows, Linux, and Web. We used the web application platform to develop our web-based dose calculator. We created two versions of the web interface for web browsers on a personal computer and a smart phone to account for differences in screen size. The web application was deployed through Xojo Cloud hosting, which was connected to the domain name http://dentaldose.org.  Figure 1 shows the workflow of the web program, where the user input data and output data are described.

## 3. Results

We tabulated technical parameters and doses for a total of 4, 18, and 51 dose sets for intraoral (Table 2), panoramic (Table 3), and CBCT (Table 4) procedures, respectively.

### 3.1. Technical Parameters and Methods Used in Dosimetry Studies

About 56% (*n* = 57) of the dose sets were from measurements, and the remaining 44% (*n* = 44) were from simulation studies. In the measurement studies, different types of dosimeters were used: the thermoluminescent dosimeter (TLD) (86%), the optically stimulated luminescent dosimeter (OSLD) (7%), the Gafchromic film (5%), and the metal-oxide-silicon field-effect transistor (MOSFET) (2%). The physical head phantoms used for measurements included the Alderson Radiation Therapy (ART) phantom (Radiology Support Devices Inc., Long Beach, CA) (70%), the ATOM adult and child phantoms (CIRS, Norfolk, VA) (20%), and CDP-R1 (Chengdu Fangtuo Simulation Technology Company Limited, China) (10%). In the simulation studies, the EGS program [27] produced about 60% of all dose sets followed by MCNP [28] (22%) and PCXMC [29] (STUK, Helsinki, Finland) (18%). A variety of computational head phantoms were used for the simulation studies: in-house head phantoms developed from patient CT images (44%), the Zubal head phantom [30] (22%), the Oak Ridge National Laboratory (ORNL)-stylized phantoms (18%), and the ICRP adult phantoms [31] (16%). The tube potential for intraoral imaging ranged from 60 to 70 kVp. The panoramic and CBCT scans used 62–73 kVp and 70–120 kVp, respectively. In the case of CBCT, the width and height of the FOV ranged from about 4 to 26 cm and the area (width × height) ranged from about 15 [11] to 600 [24] cm^2^. The beam rotation angle for CBCT was between 180° and 360°. The DAP for CBCT ranged from 91 to 1080 mGy-cm^2^.

### 3.2. Organ and Effective Doses

The organ and effective doses reported in the literature are summarized in Table 5. Mean effective doses delivered by intraoral, 1.32 (0.60–2.56) *μ*Sv, and panoramic, 17.93 (3.47–75.00) *μ*Sv, procedures are about 1% and 15% of that delivered by CBCT, 121.09 (17.10–392.20) *μ*Sv. Among the three imaging modalities, the salivary glands received the greatest dose: 22.79 *μ*Gy (intraoral), 660.24 *μ*Gy (panoramic), and 2333.95 *μ*Gy (CBCT). Among the four organs of interest, the smallest dose was delivered to the bone marrow, except for intraoral where the brain received the smallest dose.

In CBCT imaging, the child phantoms tended to receive greater doses compared with the adult phantoms, except for the salivary glands and thyroid doses (Table 6). The child phantoms received about 29% greater effective dose than the adult phantoms. The bone marrow dose of the child phantoms was about 80% greater than that of the adult phantom.

The effective dose for the larger FOV in CBCT is greater than that for the smaller FOV (Table 7). The effective dose for the large FOV (greater than 150 cm^2^) is about 1.6 times greater than that for the small FOV (less than 50 cm^2^). The brain dose for the large FOV is about eight times greater than that for the small FOV.

Table 8 shows the age- and FOV size-dependent organ and effective doses. A similar trend by age group shown in Table 6 (child's dose is greater than adult's dose) and by FOV size shown in Table 7 (large FOV gives greater dose than small FOV) is also observed.

### 3.3. Monte Carlo Dose Distribution

**Figure 2:**
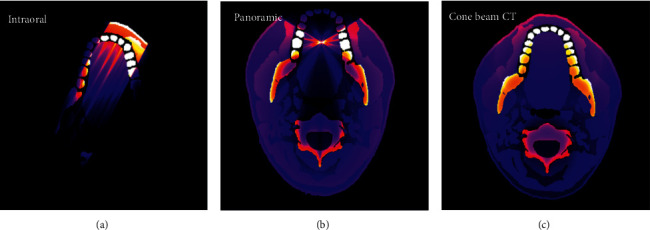
Dose distribution at the level of the lower teeth generated by MC-GPU simulations for (a) intraoral radiography, (b) panoramic radiography, and (c) cone-beam computed tomography.

2D dose distribution at the level of the center of the lower teeth for intraoral, panoramic, and CBCT calculated by MC-GPU simulations are presented in Figure 2. The angles of radiation incidence to the head phantom used in the simulations (Table 1) are visible on the head anatomy: 30° from the patient's front for intraoral; 240° rotation behind the patient's head for panoramic; and 360° rotation for CBCT. Movie clips presenting a rotating 3D dose distribution for panoramic and CBCT were created and included in the web dose calculator.

**Figure 3:**
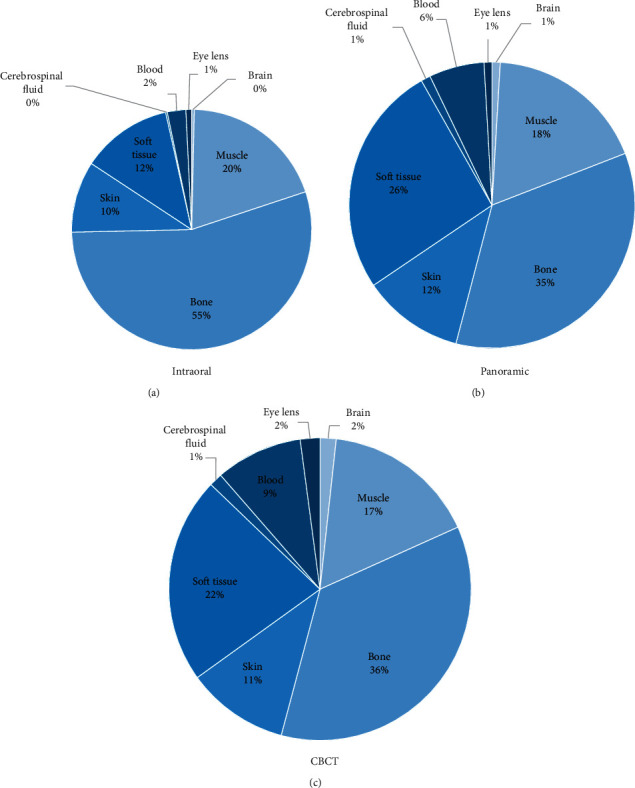
Percent fraction of radiation dose for the different tissues in the head derived from the MC-GPU simulation of the human head phantom exposed to (a) intraoral radiography, (b) panoramic radiography, and (c) cone-beam computed tomography.

The fraction of dose in different tissues (brain, muscle, bone, skin, soft tissue, cerebrospinal, blood, and eye lens) out of the total dose for intraoral, panoramic, and CBCT is shown in Figure 3. A larger portion of the radiation dose is delivered to the bone (55%) in intraoral imaging compared with panoramic and CBCT, each of which contributes about 35% of the total radiation dose to the bone. The dose delivered to the brain is nearly zero in intraoral but slightly increased to 1% in panoramic and 2% in CBCT. The dose delivered to skin and soft tissue remarkably increases from 12% (soft tissue) and 10% (skin) in intraoral to 26% (soft tissue) and 12% (skin) in panoramic and 22% (soft tissue) and 11% (skin) in CBCT.

### 3.4. Web Dose Calculator

**Figure 4:**
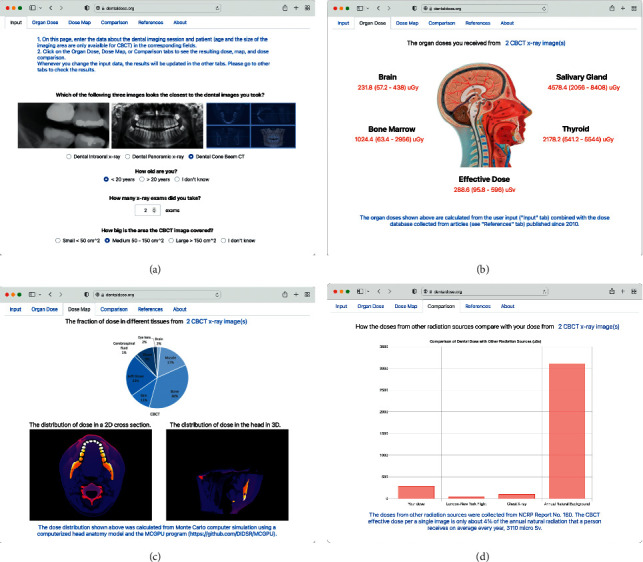
Web interfaces for (a) user input, (b) organ (brain, salivary gland, bone marrow, thyroid) and effective doses, (c) the fraction of dose in different tissues and 2D and 3D dose distributions, and (d) dose comparison with other radiation sources.

A user-friendly interactive web program was developed for a user to input the following: the type of imaging procedure, the number of image sets, age group, and FOV size (Figure 4(a)). The web interface displays organ and effective doses (Figure 4(b)), dose fraction in tissues and 2D and 3D dose distributions in the head (Figure 4(c)), and dose comparison with other radiation sources (Figure 4(d)).

## 4. Discussion

Dental imaging is one of the most common radiological imaging procedures. Although the dose level is known to be relatively low, it is still important to monitor the trend of dental dose in different dental imaging modalities. We evaluated the radiation dose received from dental imaging practices by extracting data from literature published after 2010. To efficiently present the results of the study, an interactive web-based dose calculator was created.

We compared our results from intraoral imaging with those published by Fontana et al. [32], which report the dose to the brain, salivary gland, and thyroid delivered by imaging conducted from 1940 to 2009, with the increment of ten years. To simplify the comparison, we averaged their doses in three time periods: 1940–1969, 1970–1989, and 1990–2009. The period 2010–2020 adopted in our study follows the end of their study period. A clear dose reduction was observed in the brain dose by period. Compared with the organ doses reported for the earliest period (1940–1969), the doses to the brain, salivary gland, and thyroid resulted from our study were smaller by 77%, 93%, and 93%, respectively. Compared with the latest period, 1990–2009, in Fontana et al., our organ doses were smaller by 7%, 64%, and 62% for the brain, salivary gland, and thyroid, respectively. The dose reduction may be due to the change in technical parameters and the improvement in imaging quality with the same amount of radiation.

The average effective dose from CBCT, delivering the greatest dose compared with intraoral and panoramic, was more than 92 times greater than that from intraoral and seven times greater than that from panoramic (Table 4). However, the maximum CBCT effective dose, with a large FOV, for children, 392.2 *μ*Sv [5], is about 13% of the dose from the natural radiation that a person receives on average every year, 3110 *μ*Sv [9], disregarding the radiation received from occupations and medical procedures.

We are aware of the following limitations in the current study. First, without dose calibration using measurements from clinical machines, absolute doses could not be estimated with our Monte Carlo simulations, so only relative dose distributions were obtained and analyzed. Future work may involve accurate dose measurements to provide absolute doses for a comprehensive library of technical parameters for panoramic and CBCT procedures. Second, we found that pediatric dose data were relatively limited in literature compared with those of adults, so we grouped age-dependent dose data into pediatric (age <20) and adult (age ≥ 20) for CBCT only,for which a total of 22 pediatric data points were available. Since a clear age dependency for those limited data points was not observed, possibly due to large variability, we categorized the pediatric ages into a single group. Considering the higher potential risk in pediatric patients, due to increased radiosensitivity and longer expected life span after the irradiation event, it is important to more accurately evaluate the doses delivered to them once additional dose data are available in the future. Lastly, our literature search was limited to one bibliographic database, PubMed, to the keywords we defined, and to the papers written in English.

## 5. Conclusion

A comprehensive table of the organ and effectives doses delivered by intraoral, panoramic, and CBCT dental imaging procedures was established from previously published articles collected from PubMed. We found that organ and effective doses from intraoral and panoramic radiography are substantially smaller than those from CBCT, and the maximum CBCT effective dose is about 13% of the dose from annual natural radiation. Our dose summary should be useful for comparison among doses from different dental imaging methods as well as comparison with doses from other radiation sources. The user-friendly, interactive web application (http://dentaldose.org) allows for receiving user input and displaying doses, dose distribution maps, and dose comparison with other radiation sources.

## Figures and Tables

**Table 1:** Technical parameters for intraoral, panoramic, and cone-beam computed tomography collected from literature, which were used for Monte Carlo simulations conducted using MC-GPU to estimate dose distribution within the head.

Parameters	Intraoral	Panoramic	Cone-beam computed tomography
X-ray energy (kVp)	60	73	90
Filtration (mm Al)	3.5	2.5	2.8
FOV (cm^2^)	4 × 3	10 × 0.2	10 × 10
SRD^1^ (cm)	75	35	50
Rotation angle per view (degree)	0^2^	240	360
Number of views per acquisition	1	9153	360
Number of X-rays per simulated view	10^11^	5 × 10^7^	5 × 10^9^
Total simulation time (min)^3^	33	143	760

^1^Source-to-rotation axis distance. ^2^The rectangular field was rotated 30° cranially. ^3^Simulation run in an NVIDIA GeForce GTX 1080 GPU.

**Table 2:** Dosimetry method, machine model, phantom age, technical parameters, and effective and organ (bone marrow, brain, salivary gland, and thyroid) doses for intraoral imaging procedures. No simulation-based doses are reported.

Method	Machine model	Age	Dosimeter	Phantom	Imaging protocols	DAP (mGy × cm^2^)	kVp	FOV width (cm)	FOV height (cm)	Effective dose (uSv)	Organ dose (uGy)				Reference
											Bone marrow	Brain	Salivary gland	Thyroid	
Measurement	Gendex Oralix DC	35	TLD	ART	Bitewing single image		60	3.5	4.5	0.85	0.50	0.00	27.00	2.00	Granlund et al. 2015 [10]
Measurement	Gendex Oralix DC	35	TLD	ART	Full-mouth single image		60	3.5	4.5	0.83	0.33	0.00	25.11	2.94	Granlund et al. 2015 [10]
Measurement	Prostyle	10	Gafchromic film	ATOM 706C	Periapical lateral	7.42	66	4.5	5.5	0.60	1.20	0.30	5.50	0.00	Kadesjo et al. 2018 [11]
Measurement	Prostyle	10	Gafchromic film	ATOM 706C	Periapical central	7.42	66	4.5	5.5	0.70	0.00	0.00	0.01	0.00	Kadesjo et al. 2018 [11]
Measurement	Focus	35	TLD	CDP-R1	Maxillary premolar left		70	4.5	3.5	2.56	0.55	6.12	45.25	24.47	Li et al. 2020 [12]
Measurement	Focus	35	TLD	CDP-R1	Maxillary premolar left		70	4.5	3.5	2.39	1.19	7.76	33.88	18.38	Li et al. 2020 [12]

DAP, dose area product; FOV, field of view.

**Table 3:** Dosimetry methods, machine model, phantom age, technical parameters, and effective and organ (bone marrow, brain, salivary gland, and thyroid) doses for panoramic imaging procedures.

Method	Machine model	Age (year)	Dosimeter/MC code	Phantom	Imaging protocols	kVp	Effective dose (uSv)	Organ dose (uGy)				Reference
								Bone marrow	Brain	Salivary gland	Thyroid	
Measurement	AZ3000	35	TLD	RANDO	Temporomandibular	70	11.00	3.30	29.50	549.90	29.80	Matsuo et al. 2011 [13]
Measurement	OP-200	35	TLD	ART		66	10.73	9.94	10.03	311.78	27.89	Han et al. 2013 [14]
Measurement	ORTHOPHOS CD	35	TLD	ART		71	14.33	10.74	9.41	419.17	67.87	Han et al. 2013 [14]
Measurement	ORTHOPHOS XG Plus	35	TLD	ART		69	19.06	15.99	18.41	604.05	54.60	Han et al. 2013 [14]
Measurement	ProMax	35	TLD	ART		66	26.26	14.53	15.12	939.55	54.95	Han et al. 2013 [14]
Measurement	Cranex Tome Ceph	35	TLD	ART	Jaw	66	19.00	11.00	26.00	1028.00	40.00	Granlund et al. 2015 [10]
Measurement	Cranex Tome Ceph	35	TLD	ART	Dental	66	22.00	10.00	10.00	1182.00	48.00	Granlund et al. 2015 [10]
Measurement	Veraviewepocs	35	TLD	ART	Jaw	66	23.00	13.00	10.00	869.00	53.00	Granlund et al. 2015 [10]
Measurement	Veraviewepocs	35	TLD	ART	Dental	66	30.00	18.00	5.00	939.00	52.00	Granlund et al. 2015 [10]
Measurement	Scanora	35	TLD	ART	Jaw	66	75.00	54.00	18.00	2887.00	126.00	Granlund et al. 2015 [10]
Measurement	Scanora	35	TLD	ART	Dental	66	49.00	38.00	19.00	2428.00	111.00	Granlund et al. 2015 [10]
Measurement	OP-200	5	TLD	ATOM 705	Long collimator P1	66	11.40	1.90	43.00	94.00	37.00	Davis 2015 [15]
Measurement	OP-200	5	TLD	ATOM 705	Short collimator P2	66	7.70	1.50	19.00	103.00	30.00	Davis et al. 2015 [15]
Measurement	OP-100	35	TLD	RANDO		73	7.15	5.00	72.00	109.00	24.00	Lee et al. 2016 [16]
Simulation	OP-100	35	PCXMC	ORNL		73	9.39	10.00	9.00	299.00	20.00	Lee et al. 2016 [16]
Measurement	ProMax 2D	10	Gafchromic film	ATOM 706C		62	4.10	1.80	0.00	160.00	17.00	Kadesjo et al. 2018 [11]
Measurement	ORTHOPHOSXG/Cep	35	TLD	RANDO		64	13.00	24.00	37.00	622.00	256.00	Qiang et al. 2019 [17]
Measurement	OP-100	5	TLD	ATOM 705-D		66	3.85	38.33	55.64	54.40	35.85	Lee et al. 2019 [18]
Simulation	OP-100	5	PCXMC	ORNL		66	3.47	10.51	20.00	4.32	13.29	Lee et al. 2019 [18]
Measurement	PP1	35	TLD	CDP-R1	Maxillofacial	73	8.15	8.08	33.10	113.30	54.31	Li et al. 2020 [12]
Measurement	PP1	35	TLD	CDP-R1	Maxillofacial	73	8.99	8.34	29.59	148.55	63.90	Li et al. 2020 [12]

**Table 4:** Dosimetry methods, machine model, phantom age, technical parameters, and effective and organ (bone marrow, brain, salivary gland, and thyroid) doses for cone-beam computed tomography procedures.

Method	Machine model	Age	Dosimeter/MC code	Phantom	Rotation (degree)	Imaging protocols	DAP (mGy × cm^2^)	kVp	FOV width (cm)	FOV height (cm)	Effective dose (uSv)	Organ dose (uGy)				Reference
												Bone marrow	Brain	Salivary gland	Thyroid	
Measurement	Galileos	35	TLD	ART		Maxillofacial		85	15	15	84.00	82.00	124.00	2104.00	380.00	Pauwels et al. 2012 [19]
Measurement	i-CAT	35	TLD	ART		Maxillofacial		120	16	13	83.00	116.00	375.00	1830.00	355.00	Pauwels et al. 2012 [19]
Measurement	Iluma Elite	35	TLD	ART		Maxillofacial		120	21	14	368.00	660.00	3415.00	7225.00	1230.00	Pauwels et al. 2012 [19]
Measurement	Kodak 9500	35	TLD	ART		Maxillofacial		90	20	18	136.00	206.00	1205.00	2676.00	585.00	Pauwels et al. 2012 [19]
Measurement	NewTom VG	35	TLD	ART		Maxillofacial		110	15	10	83.00	115.00	251.00	1690.00	354.00	Pauwels et al. 2012 [19]
Measurement	NewTom VGi	35	TLD	ART		Maxillofacial		110	15	15	194.00	186.00	605.00	2855.00	2045.00	Pauwels et al. 2012 [19]
Measurement	Scanora 3D	35	TLD	ART		Maxillofacial		85	14.5	13.5	68.00	86.00	255.00	1568.00	296.00	Pauwels et al. 2012 [19]
Measurement	SkyView	35	TLD	ART		Maxillofacial		90	17	17	87.00	134.00	719.00	1582.00	474.00	Pauwels et al. 2012 [19]
Measurement	3D Accuitomo 170	35	TLD	ART		Maxilla		90	10	5	54.00	112.00	189.00	2138.00	148.00	Pauwels et al. 2012 [19]
Measurement	i-CAT NG	35	TLD	ART		Mandible		120	16	6	45.00	33.00	46.00	973.00	251.00	Pauwels et al. 2012 [19]
Measurement	Kodak 9500	35	TLD	ART		Dentoalveolar		90	15	8	92.00	85.00	91.00	2166.00	541.00	Pauwels et al. 2012 [19]
Measurement	NewTom VGi	35	TLD	ART		Dentoalveolar		110	12	8	265.00	294.00	431.00	6372.00	1293.00	Pauwels et al. 2012 [19]
Measurement	Picasso trio	35	TLD	ART		Dentoalveolar		85	12	7	123.00	126.00	134.00	2982.00	551.00	Pauwels et al. 2012 [19]
Measurement	Picasso trio	35	TLD	ART		Dentoalveolar		85	12	7	81.00	62.00	39.00	1837.00	583.00	Pauwels et al. 2012 [19]
Measurement	ProMax 3D	35	TLD	ART		Dentoalveolar		84	8	8	122.00	88.00	53.00	2576.00	1021.00	Pauwels et al. 2012 [19]
Measurement	ProMax 3D	35	TLD	ART		Dentoalveolar		84	8	8	28.00	27.00	28.00	596.00	202.00	Pauwels et al. 2012 [19]
Measurement	Scanora 3D	35	TLD	ART		Dentoalveolar		85	10	7.5	46.00	42.00	45.00	1285.00	148.00	Pauwels et al. 2012 [19]
Measurement	Scanora 3D	35	TLD	ART		Mandible		85	10	7.5	47.00	34.00	25.00	1052.00	352.00	Pauwels et al. 2012 [19]
Measurement	Scanora 3D	35	TLD	ART		Maxilla		85	10	7.5	45.00	37.00	31.00	1117.00	240.00	Pauwels et al. 2012 [19]
Measurement	Veraviewepocs 3D	35	TLD	ART		Dentoalveolar		70	8	8	73.00	55.00	40.00	1956.00	330.00	Pauwels et al. 2012 [19]
Measurement	3D Accuitomo 170	35	TLD	ART		Lower jaw, molar region		90	4	4	43.00	37.00	37.00	2120.00	195.00	Pauwels et al. 2012 [19]
Measurement	Kodak 9000 3D	35	TLD	ART		Upper jaw, front region		70	5	3.7	19.00	21.00	18.00	523.00	30.00	Pauwels et al. 2012 [19]
Measurement	Kodak 9000 3D	35	TLD	ART		Lower jaw, molar region		70	5	3.7	40.00	78.00	290.00	709.00	251.00	Pauwels et al. 2012 [19]
Measurement	Pax-Uni3D	35	TLD	ART		Upper jaw, front region		85	5	5	44.00	47.00	28.00	1073.00	209.00	Pauwels et al. 2012 [19]
Measurement	ProMax 3D	35	MOSFET	RANDO RAN102	200		574		8	8	153.00	25.00	4.30	32.00	32.00	Koivisto et al. 2012 [20]
Simulation	ProMax 3D	35	PCXMC	ORNL Phantom	200		574		8	8	131.00	6.90	2.80	41.00	8.40	Koivisto et al. 2012 [20]
Simulation	i-CAT	35	EGS4	ICRP ADULT	360		556		16	13	66.00	50.00	590.00	1270.00	80.00	Morant et al. 2013 [21]
Simulation	i-CAT	35	EGS4	ICRP ADULT	360		476		16	11	58.00	40.00	310.00	1230.00	80.00	Morant et al. 2013 [21]
Simulation	i-CAT	35	EGS4	ICRP ADULT	360		415		16	10	53.00	30.00	190.00	1150.00	70.00	Morant et al. 2013 [21]
Simulation	Accuitomo 170	5	EGSnrc	In-house	360	Standard resolution		90	6	6	172.26	1052.00	89.00	2965.00	387.00	Stratis et al. 2016 [22]
Simulation	Accuitomo 170	5	EGSnrc	In-house	360	Standard resolution		90	8	8	297.97	1478.00	181.00	4204.00	1919.00	Stratis et al. 2016 [22]
Simulation	Accuitomo 170	8	EGSnrc	In-house	360	Standard resolution		90	6	6	119.35	518.00	92.00	2623.00	751.00	Stratis et al. 2016 [22]
Simulation	Accuitomo 170	8	EGSnrc	In-house	360	Standard resolution		90	8	8	228.32	986.00	181.00	3831.00	1747.00	Stratis et al. 2016 [22]
Simulation	Accuitomo 170	12	EGSnrc	In-house	360	Standard resolution		90	6	6	111.76	607.00	42.00	1862.00	497.00	Stratis et al. 2016 [22]
Simulation	Accuitomo 170	12	EGSnrc	In-house	360	Standard resolution		90	8	8	250.95	913.00	90.00	2961.00	2772.00	Stratis et al. 2016 [22]
Simulation	Accuitomo 170	35	EGSnrc	ICRP AF	360	Standard resolution		90	6	6	81.54	376.00	112.00	2830.00	175.00	Stratis et al. 2016 [22]
Simulation	Accuitomo 170	35	EGSnrc	ICRP AF	360	Standard resolution		90	8	8	126.68	565.00	224.00	4308.00	339.00	Stratis et al. 2016 [22]
Simulation	i-CAT	35	PCXMC	ORNL Phantom	360			120	16	13	30.99	42.90	269.58	738.29	46.97	Yeh and Chen 2018 [23]
Simulation	ProMax 3D	5	EGSnrc	In-house		Canine XS/ND		96	4.2	5.5	134.90	200.00	152.00	3558.00	214.00	Marcu et al. 2018 [24]
Simulation	ProMax 3D	5	EGSnrc	In-house		Molars XS/ND		96	4.2	5.5	155.90	222.00	170.00	4352.00	256.00	Marcu et al. 2018 [24]
Simulation	ProMax 3D	5	EGSnrc	In-house		Molars XS/ND		96	5	5.5	220.20	23.80	15.80	336.30	67.90	Marcu et al. 2018 [24]
Simulation	ProMax 3D	5	EGSnrc	In-house		Molars XS/ND		96	10	9	68.60	77.70	62.70	1400.60	315.30	Marcu et al. 2018 [24]
Simulation	ProMax 3D	5	EGSnrc	In-house		Molars XS/ND		96	10	5.5	55.70	45.00	28.60	1211.00	308.80	Marcu et al. 2018 [24]
Simulation	ProMax 3D	5	EGSnrc	In-house		Molars XS/ND		96	13	16	75.60	149.60	948.20	1453.60	206.60	Marcu et al. 2018 [24]
Simulation	ProMax 3D	5	EGSnrc	In-house		S/ND		96	23	26	178.40	614.00	4324.00	1596.00	2677.00	Marcu et al. 2018 [24]
Simulation	ProMax 3D	8	EGSnrc	In-house		Canine XS/ND		96	4.2	5.5	124.60	85.00	126.00	3080.00	157.00	Marcu et al. 2018 [24]
Simulation	ProMax 3D	8	EGSnrc	In-house		Molars XS/ND		96	4.2	5.5	128.60	93.00	141.00	3458.00	187.00	Marcu et al. 2018 [24]
Simulation	ProMax 3D	8	EGSnrc	In-house		Molars XS/ND		96	5	5.5	17.10	15.20	14.40	419.00	38.60	Marcu et al. 2018 [24]
Simulation	ProMax 3D	8	EGSnrc	In-house		Molars XS/ND		96	10	9	60.80	54.30	48.70	1389.00	290.90	Marcu et al. 2018 [24]
Simulation	ProMax 3D	8	EGSnrc	In-house		Molars XS/ND		96	10	5.5	47.90	31.70	219.00	1028.00	270.60	Marcu et al. 2018 [24]
Simulation	ProMax 3D	8	EGSnrc	In-house		Molars XS/ND		96	13	16	64.70	104.70	772.20	1319.00	202.60	Marcu et al. 2018 [24]
Simulation	ProMax 3D	8	EGSnrc	In-house		S/ND		96	23	26	392.20	518.00	4247.00	4007.00	2908.00	Marcu et al. 2018 [24]
Simulation	ProMax 3D	35	EGSnrc	ICRP AM				96	23	26	256.10	362.80	4653.00	4378.00	294.00	Marcu et al. 2018 [24]
Simulation	Cranex3Dx	35	MCNP6.1	ZUBAL Head	180	Maxillary first molar/SR	168	90	5	5	40.00	375.00	105.00	1154.00	139.00	Kralik et al. 2018 [25]
Simulation	Cranex3Dx	35	MCNP6.1	ZUBAL Head	180	Mandibular dental arch/SR	280	90	6.1	7.8	75.00	370.00	55.00	1278.00	1252.00	Kralik et al. 2018 [25]
Simulation	Cranex3Dx	35	MCNP6.1	ZUBAL Head	180	Both dental arches	338	90	7.8	7.8	74.00	578.00	1155.00	2045.00	535.00	Kralik et al. 2018 [25]
Simulation	Cranex3Dx	35	MCNP6.1	ZUBAL Head	180	Mandible/SR	528	90	7.8	15	142.00	564.00	96.00	2275.00	2465.00	Kralik et al. 2018 [25]
Simulation	Cranex3Dx	35	MCNP6.1	ZUBAL Head	180	Viscerocranium/SR	755	90	13	15	170.00	1034.00	552.00	2847.00	2370.00	Kralik et al. 2018 [25]
Simulation	Cranex3Dx	35	MCNP6.1	ZUBAL Head	360	Maxillary first molar/SR	168	90	5	5	37.00	335.00	78.00	592.00	104.00	Kralik et al. 2018 [25]
Simulation	Cranex3Dx	35	MCNP6.1	ZUBAL Head	360	Mandibular dental arch/SR	280	90	6.1	7.8	105.00	400.00	49.00	1161.00	789.00	Kralik et al. 2018 [25]
Simulation	Cranex3Dx	35	MCNP6.1	ZUBAL Head	360	Both dental arches	338	90	7.8	7.8	92.00	621.00	153.00	1559.00	789.00	Kralik et al. 2018 [25]
Simulation	Cranex3Dx	35	MCNP6.1	ZUBAL Head	360	Mandible/SR	528	90	7.8	15	184.00	607.00	87.00	2035.00	3533.00	Kralik et al. 2018 [25]
Simulation	Cranex3Dx	35	MCNP6.1	ZUBAL Head	360	Viscerocranium/SR	755	90	13	15	214.00	1005.00	523.00	2371.00	3384.00	Kralik et al. 2018 [25]
Measurement	ProMax 3D	10	TLD	ATOM 706C	360		510	90	4	5	88.00	130.00	510.00	1800.00	200.00	Kadesjo et al. 2018 [11]
Measurement	NewTom5G	10	TLD	ATOM 706C	360		1080	110	6	6	172.00	270.00	760.00	3800.00	340.00	Kadesjo et al. 2018 [11]
Measurement	Galileos	35	TLD	RANDO		Maxillofacial		85	15	15	203.00	278.00	636.00	7775.00	8727.00	Qiang et al. 2019 [17]
Simulation	CS9300	35	PCXMC	ORNL Phantom	360	Facial	251	90	17	13.5	160.90	180.00	460.00	4570.00	450.00	Lee et al. 2020 [26]
Simulation	CS9300	35	PCXMC	ORNL Phantom	360	Dual jaw	91	90	10	10	94.40	100.00	230.00	1950.00	580.00	Lee et al. 2020 [26]
Simulation	RAYSCAN	35	PCXMC	ORNL Phantom	360	Large jaw	177	80	16	10	198.00	200.00	220.00	5360.00	320.00	Lee et al. 2020 [26]
Simulation	RAYSCAN	35	PCXMC	ORNL Phantom	360	Jaw	91	80	10	10	195.20	190.00	280.00	4700.00	410.00	Lee et al. 2020 [26]
Measurement	CS9300	35	OSLD	ATOM 702C	360	Facial	251	90	17	13.5	181.40	20.00	1020.00	3160.00	1210.00	Lee et al. 2020 [26]
Measurement	CS9300	35	OSLD	ATOM 702C	360	Dual jaw	91	90	10	10	90.70	10.00	230.00	1950.00	580.00	Lee et al. 2020 [26]
Measurement	RAYSCAN	35	OSLD	ATOM 702C	360	Large jaw	177	80	16	10	228.50	10.00	380.00	4540.00	1680.00	Lee et al. 2020 [26]
Measurement	RAYSCAN	35	OSLD	ATOM 702C	360	Jaw	91	80	10	10	213.80	10.00	570.00	3930.00	1500.00	Lee et al. 2020 [26]
Measurement	KaVo 3D eXami	35	TLD	CDP-R1		Maxillofacial		120	14.5	8.5	56.63	15.22	427.82	1177.24	476.04	Li et al. RPD 2020 [12]
Measurement	KaVo 3D eXami	35	TLD	CDP-R1		Maxillofacial		120	14.5	8.5	55.18	11.63	494.50	1314.85	452.68	Li et al. RPD 2020 [12]

DAP, dose area product; FOV, field of view.

**Table 5:** Minimum, mean, and maximum values of effective and organ (bone marrow, brain, salivary gland, and thyroid) doses from intraoral, panoramic, and cone-beam computed tomography procedures reported in the selected publications.

Procedures		Effective dose (*μ*Sv)	Organ dose (*μ*Gy)			
			Bone marrow	Brain	Salivary gland	Thyroid
Intraoral (*n* = 6)	Mean	1.32	0.63	2.36	22.79	7.97
	Min	0.60	0.00	0.00	0.01	0.00
	Max	2.56	1.20	7.76	45.25	24.47

Panoramic (*n* = 21)	Mean	17.93	14.66	23.28	660.24	57.93
	Min	3.47	1.50	0.00	4.32	13.29
	Max	75.00	54.00	72.00	2887.00	256.00

Cone-beam computed tomography (*n* = 76)	Mean	121.09	254.78	471.65	2333.95	811.16
	Min	17.10	6.90	2.80	32.00	8.40
	Max	392.20	1478.00	4653.00	7775.00	8727.00

**Table 6:** Minimum, mean, and maximum values of effective and organ (bone marrow, brain, salivary gland, and thyroid) doses from cone-beam computed tomography procedures by age group (children and adults) reported in the selected publications.

Age group		Effective dose (*μ*Sv)	Organ dose (*μ*Gy)			
			Bone marrow	Brain	Salivary gland	Thyroid
Children (*n* = 22)	Mean	143.9	372.2	600.7	2393.3	759.7
	Min	17.1	15.2	14.4	336.3	38.6
	Max	392.2	1478.0	4324.0	4352.0	2908.0

Adults (*n* = 54)	Mean	111.8	207.0	419.1	2309.7	832.1
	Min	19.0	6.9	2.8	32.0	8.4
	Max	368.0	1034.0	4653.0	7775.0	8727.0

**Table 7:** Minimum, mean, and maximum values of effective and organ (bone marrow, brain, salivary gland, and thyroid) doses from cone-beam computed tomography procedures by field-of-view area (FOV width × height, cm^2^) reported in the selected publications.

FOV area (cm^2^)		Effective dose (*μ*Sv)	Organ dose (*μ*Gy)			
			Bone marrow	Brain	Salivary gland	Thyroid
Small (<50) (*n* = 20)	Mean	96.5	262.8	144.2	1984.7	312.0
	Min	17.1	15.2	14.4	336.3	38.6
	Max	220.2	1052.0	760.0	4352.0	1252.0

Medium (50–150) (*n* = 32)	Mean	113.7	246.4	184.9	2137.2	780.7
	Min	28.0	6.9	2.8	32.0	8.4
	Max	298.0	1478.0	1155.0	4700.0	3533.0

Large (>150) (*n* = 24)	Mean	151.4	259.3	1126.8	2887.3	1267.7
	Min	31.0	10.0	124.0	738.3	47.0
	Max	392.2	1034.0	4653.0	7775.0	8727.0

**Table 8:** Minimum, mean, and maximum values of effective and organ (bone marrow, brain, salivary gland, and thyroid) doses from cone-beam computed tomography procedures by age group (children and adults) and field-of-view area (FOV width × height, cm^2^) reported in the selected publications.

FOV area (cm^2^) age group			Effective dose (*μ*Sv)	Organ dose (*μ*Gy)			
				Bone marrow	Brain	Salivary gland	Thyroid
Small (<50)	Children (*n* = 11)	Mean	131.3	292.4	192.0	2568.5	281.4
		Min	17.1	15.2	14.4	336.3	38.6
		Max	220.2	1052.0	760.0	4352.0	751.0
	Adults (*n* = 9)	Mean	53.8	226.6	85.8	1271.1	349.3
		Min	19.0	21.0	18.0	523.0	30.0
		Max	105.0	400.0	290.0	2830.0	1252.0

Medium (50–150)	Children (*n* = 7)	Mean	144.3	512.2	115.9	2289.2	1089.1
		Min	47.9	31.7	28.6	1028.0	270.6
		Max	298.0	1478.0	219.0	4204.0	2772.0
	Adults (*n* = 25)	Mean	105.2	172.0	204.3	2094.7	694.4
		Min	28.0	6.9	2.8	32.0	8.4
		Max	265.0	621.0	1155.0	6372.0	3533.0

Large (>150)	Children (*n* = 4)	Mean	177.7	346.6	2572.9	2093.9	1498.6
		Min	64.7	104.7	772.2	1319.0	202.6
		Max	392.2	614.0	4324.0	4007.0	2908.0
	Adults (*n* = 20)	Mean	146.1	241.9	837.6	3046.0	1221.5
		Min	31.0	10.0	124.0	738.3	47.0
		Max	368.0	1034.0	4653.0	7775.0	8727.0

## Data Availability

The radiation dose data from dental imaging procedures used to support the findings of this study are included within the article.
